# Whole-genome sequencing of 13 Arctic plants and draft genomes of *Oxyria digyna* and *Cochlearia groenlandica*

**DOI:** 10.1038/s41597-024-03569-6

**Published:** 2024-07-18

**Authors:** Jun Kim, Jiseon Lim, Moonkyo Kim, Yoo Kyung Lee

**Affiliations:** 1 Department of Convergent Bioscience and Informatics, College of Bioscience and Biotechnology, Chungnam National University, Daejeon, 34134 Korea; 2 Korea Polar Research Institute, Incheon, 21990 Korea; 3 Department of Life Sciences, Incheon National University, Incheon, 22012 Korea; 4 Department of Polar Sciences, University of Science and Technology, Incheon, 21990 Korea

**Keywords:** DNA sequencing, Plant ecology

## Abstract

To understand the genomic characteristics of Arctic plants, we generated 28–44 Gb of short-read sequencing data from 13 Arctic plants collected from the High Arctic Svalbard. We successfully estimated the genome sizes of eight species by using the *k*-mer-based method (180–894 Mb). Among these plants, the mountain sorrel (*Oxyria digyna*) and Greenland scurvy grass (*Cochlearia groenlandica*) had relatively small genome sizes and chromosome numbers. We obtained 45 × and 121 × high-fidelity long-read sequencing data. We assembled their reads into high-quality draft genomes (genome size: 561 and 250 Mb; contig N50 length: 36.9 and 14.8 Mb, respectively), and correspondingly annotated 43,105 and 29,675 genes using ~46 and ~85 million RNA sequencing reads. We identified 765,012 and 88,959 single-nucleotide variants, and 18,082 and 7,698 structural variants (variant size ≥ 50 bp). This study provided high-quality genome assemblies of *O*. *digyna* and *C*. *groenlandica*, which are valuable resources for the population and molecular genetic studies of these plants.

## Background & Summary

Arctic plants live in vulnerable environments. They are exposed to short growing seasons, temperature fluctuations, strong winds, and oligotrophic soils^1^. Occasionally, their habitats are disturbed by an overflow of glacial meltwater^2^. The formation of thaw ponds by permafrost thawing and changes in temperature and precipitation results in vegetation shifts and/or community trait changes^3,4^. Arctic plants are driven into competition with subarctic plants and are influenced by boreal animals, such as moose, beaver, red fox, and boreal birds that expand into the Arctic tundra^5^. Before the population of plants of the Arctic tundra is endangered, investigating the legacy of their adaptation to harsh Arctic environments merits understanding. However, little is known about the genomes of Arctic plants. Therefore, we attempted to obtain whole-genome sequencing data for 13 Arctic plants commonly found in Svalbard, using Illumina sequencing technology.

*Oxyria digyna* is a fast-growing forb that is widely distributed in the Arctic and alpine regions^6–8^. This plant is an important food source for herbivores, such as insects, birds, mammals, and even indigenous people in the Arctic^6,9–11^. Phylogeographic analysis of the plastid and nuclear genes showed that *O*. *digyna* originated in the Qinghai-Tibet Plateau and spread to Russia, eastward to North America, and westward to Western Europe^8^.

Despite its wide geographic distribution, only two major ecotypes of *O*. *digyna*, namely northern and southern, have been well-characterized. Both are long-day plants, and the northern type has fewer flowering branches and more rhizomes than the southern type^12,13^. As no reference genome sequence is available for this species, the genetic architecture underlying phenotypic variations and other population genetic structures has not yet been fully resolved.

*Cochlearia* is a genus comprising approximately 30 species, including annual and perennial herbs of the family Brassicaceae. The leaves are smoothly rounded or kidney-shaped and have long stalks that resemble spoons^14^. The scientific name *Cochlearia* derives from the Greek “kokhliárion” meaning a spoon and the English name of *Cochlearia* is also spoonwort. Some *Cochlearia* plants contain enough vitamin C and another common name, “scurvy-grass,” reflects its use as a traditional remedy for scurvy, a disease caused by the deficiency of vitamin C. Salt and heavy metal tolerance has been reported in some *Cochlearia* plants^15^.

*Cochlearia groenlandica* is a biennial, occasionally short-lived, perennial herb with a wide distribution in Greenland, Svalbard, Iceland, Alaska, Canada, and Russia^16^. It grows in various habitats, including gravelly and sandy plains, sediment plains, moss tundra, patterned grounds, seashores, and bird-cliff meadows. It can grow relatively well regardless of the soil pH or nutrient level and can survive and reproduce under harsh conditions as a stunted form^17^. The size of individual plants and leaves varies; they can be ten times larger in nutrient-rich areas, such as below bird cliffs. Polar bears graze on *C*. *groenlandica* at the foot of a large seabird colony on a cliff in Spitsbergen, Svalbard^18^. Chromosome counting confirmed the haploid chromosome number of *C*. *groenlandica* at x = 7^19,20^. There are two populations of *C*. *groenlandica* in Iceland with genetic and morphological differences. The alpine population is genetically and morphologically similar to those in Greenland and Svalbard, whereas the coastal population is different from the Arctic population^20^. Similar to *O*. *digyna*, *C*. *groenlandica* has no reference genome or limited genomic resources, which limits the genetic understanding of this Arctic plant.

**Fig. 1:**
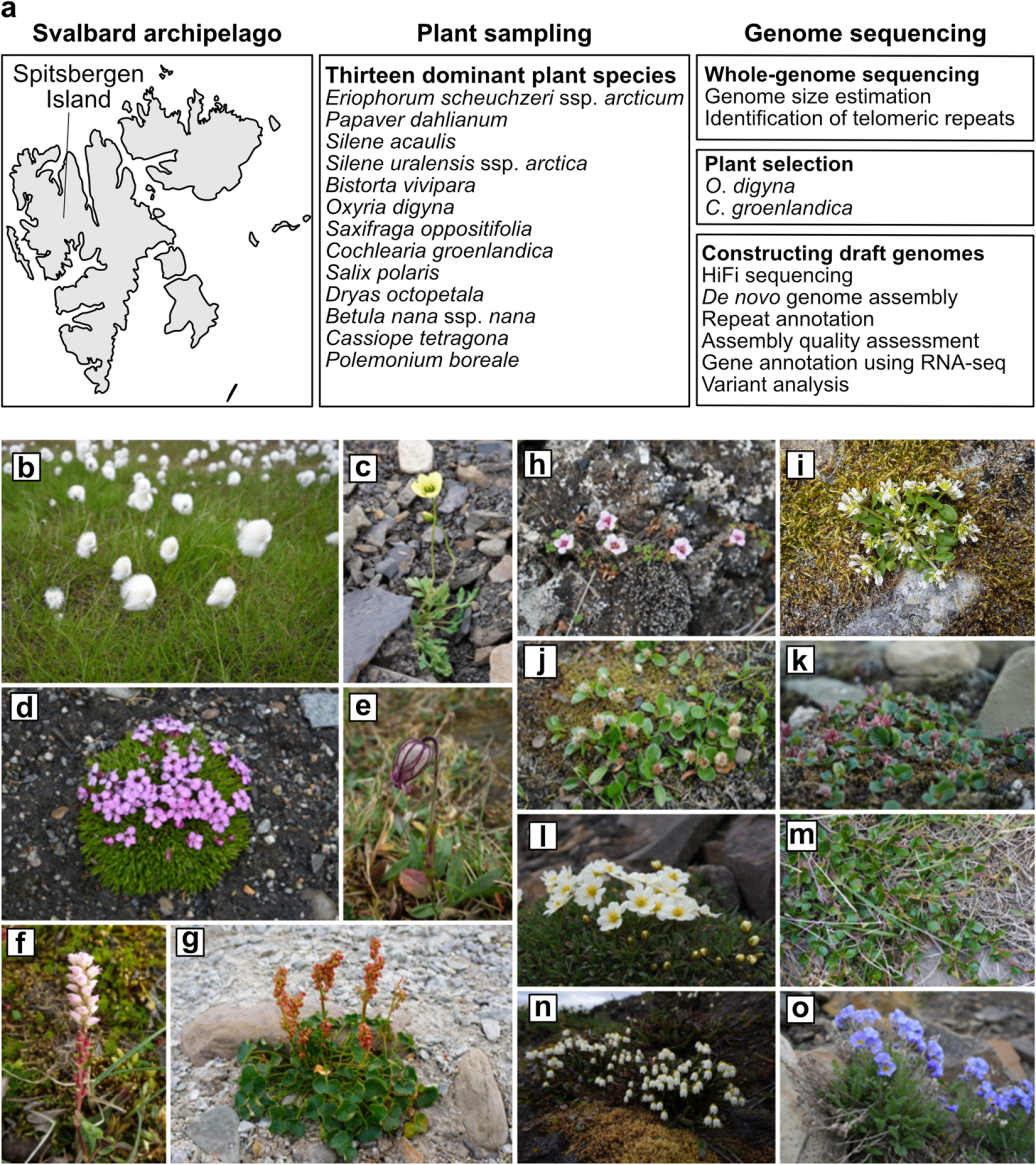
Experimental scheme and plant samples found in the Spitsbergen Island. (**a**) Sampling location and summarized experimental procedure. The thirteen species that we studied in this study are listed in the middle. Purposes and short descriptions of whole-genome sequencing experiments are listed on the right. (**b**–**o**) Thirteen Arctic plants analyzed in the study. (**b**) *Eriophorum scheuchzeri* ssp. *arcticum*; (**c**) *Papaver dahlianum*; (**d**) *Silene acaulis*; (**e**) *Silene uralensis* ssp. *arctica*; (**f**) *Bistorta vivipara*; (**g**) *Oxyria digyna*; (**h**) *Saxifraga oppositifolia*; (**i**) *Cochlearia groenlandica*; (**j**) *Salix polaris* male; (**k**) *Salix polaris* female; (**l**) *Dryas octopetala*; (**m**) *Betula nana* ssp. *nana*; (**n**) *Cassiope tetragona*; and (**o**) *Polemonium boreale*.

**Fig. 2:**
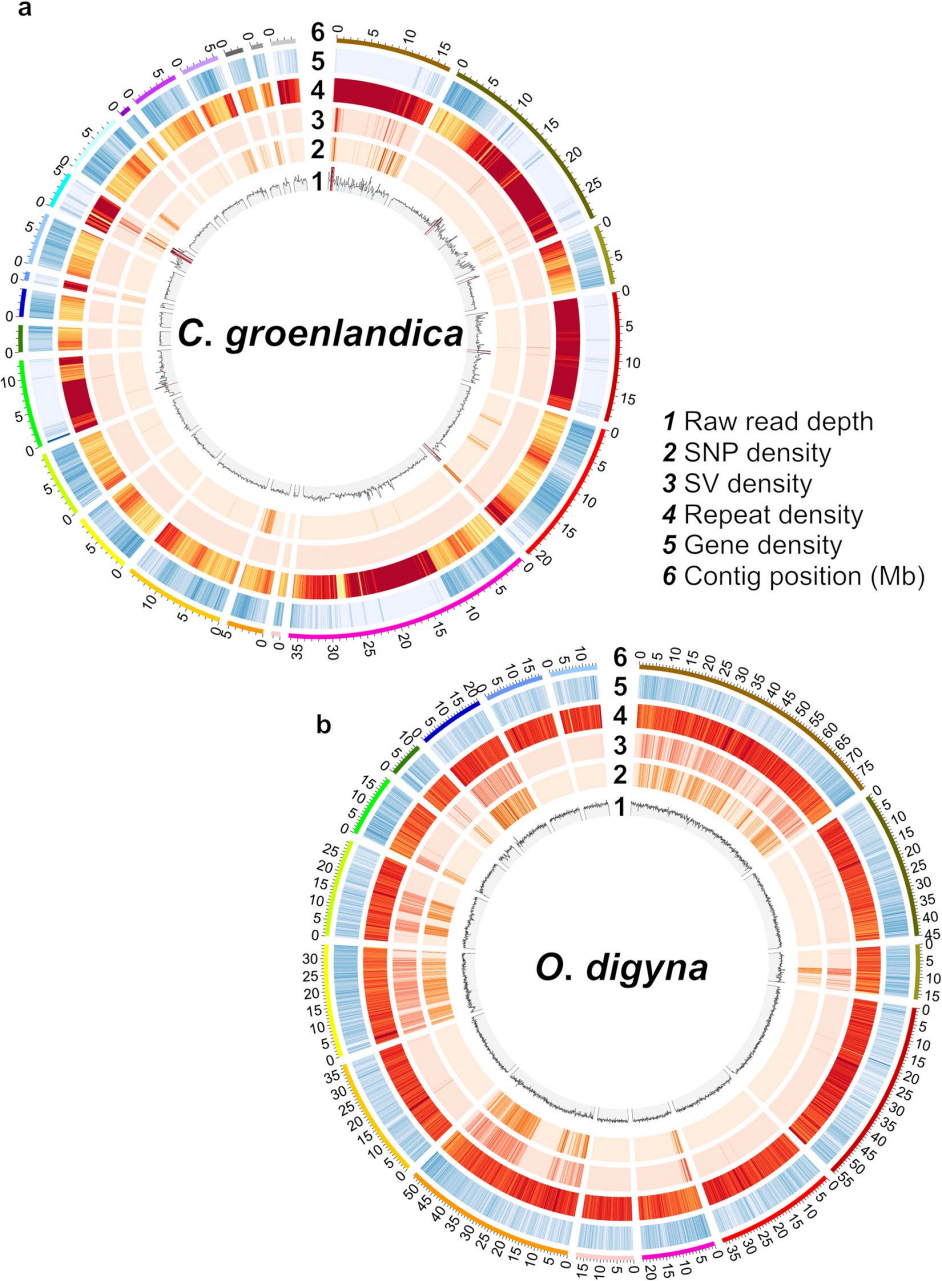
Circos plots for read depth, variant, repetitive sequence, and gene densities of (**a**) *C*. *groenlandica* and (**b**) *O*. *digyna* along contigs (**1**–**6**; 100-kb binning intervals). Lighter colours represent lower densities than darker colours. Numbers along contigs represent their positions (Mb). Read depth ranges from 0 to 230 for *C*. *groenlandica* and from 0 to 90 for *O*. *digyna* in each interval; Gene count does 0–56 and 0–30, respectively; SNP count does 0–980 and 0–1230; SV count does 0–145 and 0–33, and repetitive sequence ratio does 0–1. Only ≥ 1 Mb- or 10 Mb-sized contigs are shown for *C*. *groenlandica* and *O*. *digyna* to remove too short contigs while retaining representation for >90% of the genome.

In this study, we provide short-read sequencing data of 13 Arctic plants as well as high-quality draft genome sequences of *O*. *digyna* and *C*. *groenlandica* generated using the high-fidelity (HiFi) long-read sequencing technology of Pacific Biosciences (PacBio) (Fig. 1). We assessed the assembly quality and annotated the genetic variants, repetitive sequences, and genes in these genome assemblies (Fig. 2). These are the first draft genomes of the genera, *Oxyria* and *Cochlearia*, based on long-read sequencing data. This will be a valuable resource for understanding the genomic characteristics of Arctic plants and the population structures of *O*. *digyna* and *C*. *groenlandica*.

## Methods

### Plant sampling

Arctic plant samples were collected from Spitsbergen Island in the Svalbard Archipelago between July 25 and August 3, 2021. Thirteen dominant species in Svalbard were selected for genome size estimation, including *Eriophorum scheuchzeri* ssp. *arcticum*, *Papaver dahlianum*, *Silene acaulis*, *Silene uralensis* ssp. *arctica*, *Bistorta vivipara*, *Oxyria digyna*, *Saxifraga oppositifolia*, *Cochlearia groenlandica*, *Salix polaris*, *Dryas octopetala*, *Betula nana* ssp. *nana*, *Cassiope tetragona*, and *Polemonium boreale* (Fig. 1). Only the indicated number of leaves required for analysis (less than 2.0 g) was collected from each plant to minimize its impact on the local population. The collected samples were immediately placed in yellow envelopes and dried in the field using silica gel. The plant leaves were lyophilized in envelopes at the Dasan Arctic Research Station and stored in 15-mL conical tubes at 4 °C until they were transferred to Korea.

### DNA extraction and Illumina sequencing

DNA was extracted using the GeneAll® ExgeneTM Plant SV Mini Kit (GeneAll Biotechnology). DNA extraction was performed by slightly modifying the manufacturer protocol. The concentration and quality of the extracted DNA were checked using a NanoPhotometer® NP80 (Implen GmbH), and the DNA quality was checked by electrophoresis.

Illumina sequencing was performed by DNALink (https://dnalink.com/). The DNA library for sequencing was produced using an Illumina DNA Sample Prep Kit, and the completed DNA library was sequenced with paired-end reads of 151 bp using the Illumina NovaSeq 6000 platform. DNA sequencing data (28–44 Gb) were obtained (Table 1)^21^, which were suitable for estimating genome sizes of approximately 1 Gb.

**Table 1:** Species information and sequencing data summary of 13 Svalbard plants. ND, Not determined; NA, Not applicable; Chromosome numbers and ploidy data were obtained from Svalbard Flora (https://svalbardflora.no/).

Purpose	Scientific name	Chromosome number (Ploidy level)	Sample type	Sequencing platform	Total amount (Gb)	Read length (bp)	Estimated or assembled genome (size Mb)	Telomeric repeat
Genome size estimation	*Eriophorum scheuchzeri ssp. arcticum*	2n = 58 (ND)	DNA	Illumina NovaSeq 6000	34.59	151	306	TTTAGGG
Genome size estimation	*Papaver dahlianum*	2n = 70 (10x)	DNA	Illumina NovaSeq 6000	34.34	151	ND	TTCAGGG
Genome size estimation	*Silene acaulis*	2n = 24 (2x)	DNA	Illumina NovaSeq 6000	27.86	151	ND	TTTAGGG
Genome size estimation	*Silene uralensis ssp. arctica*	2n = 24 (2x)	DNA	Illumina NovaSeq 6000	44.30	151	894	TTTAGGG
Genome size estimation	*Bistorta vivipara*	2n = 77–132 (Polyploidy)	DNA	Illumina NovaSeq 6000	37.90	151	ND	TTTAGGG
Genome size estimation	*Oxyria digyna*	2n = 14 (2x)	DNA	Illumina NovaSeq 6000	33.65	151	352	TTTAGGG
Genome size estimation	*Saxifraga oppositifolia*	2n = 26, 39, 52 (2x, 3x, 4x)	DNA	Illumina NovaSeq 6000	32.26	151	ND	TTTTAGGG
Genome size estimation	*Cochlearia groenlandica*	2n = 14 (2x)	DNA	Illumina NovaSeq 6000	41.14	151	180	TTTAGGG
Genome size estimation	*Salix polaris*	2n = 114 (6x)	DNA	Illumina NovaSeq 6000	34.65	151	383	TTTAGGG
Genome size estimation	*Dryas octopetala*	2n = 18 (2x)	DNA	Illumina NovaSeq 6000	37.39	151	204	TTTAGGG
Genome size estimation	*Betula nana ssp. nana*	2n = 28 (2x)	DNA	Illumina NovaSeq 6000	33.64	151	689	TTTAGGG
Genome size estimation	*Cassiope tetragona*	2n = 26 (2x)	DNA	Illumina NovaSeq 6000	30.50	151	798	TTTAGGG
Genome size estimation	*Polemonium boreale*	2n = 18 (2x)	DNA	Illumina NovaSeq 6000	29.29	151	ND	TTTAGGG
Genome assembly	*Oxyria digyna*	2n = 14 (2x)	DNA	PacBio SequelII	25.51	17,289	561	TTTAGGG
Genome assembly	*Cochlearia groenlandica*	2n = 14 (2x)	DNA	PacBio SequelII	30.16	18,429	250	TTTAGGG
Gene annotation	*Oxyria digyna*	2n = 14 (2x)	RNA	Illumina NovaSeq 6000	13.98	151	NA	NA
Gene annotation	*Cochlearia groenlandica*	2n = 14 (2x)	RNA	Illumina NovaSeq 6000	25.72	151	NA	NA

### Genome size estimation

KAT (version 2.4.1) was used to estimate the genome size of 13 Svalbard plants^22^. The command *kat hist -m 27* was applied to all prepared Illumina sequencing data. The genome sizes of the eight species ranged from 180 Mb to 894 Mb (Table 1). It is noteworthy that if their genomes are highly repetitive, the true genome size could be larger than the estimated size, although the order of magnitude would be similar. We were unable to determine the genome sizes of *Bistorta vivipara*, *Papaver dahlianum*, *Polemonium boreale*, *Silene acaulis*, and *Saxifraga oppositifolia*. We suspect that the genome size of these species may have exceeded 1 Gb. Because the genome sizes of *O*. *digyna* and *C*. *groenlandica* were estimated to be relatively small, ~352 and ~180 Mb, respectively, and their haploid (n) chromosome numbers were only seven, we focused on constructing the draft genomes of these plant species.

### Identification of telomeric repeats

We also attempted to annotate the telomeric repeats of each species using the short-read sequencing data, because telomeric repeats can be changed in plants and other species-rich phylums^23–33^. As telomeric repeats should be found as sequential and continuous repetitive sequences in a single read, we subsampled 20 million reads from the Illumina reads of the 13 Svalbard plants and only used 60-bp regions of each read by trimming off the initial 10 bp and the last 81 bp. We then counted *k*-mers of sequentially repetitive units that were similar to the canonical plant telomeric repeat, TTTAGGG (*Arabidopsis*-type)^26^.

**Fig. 3:**
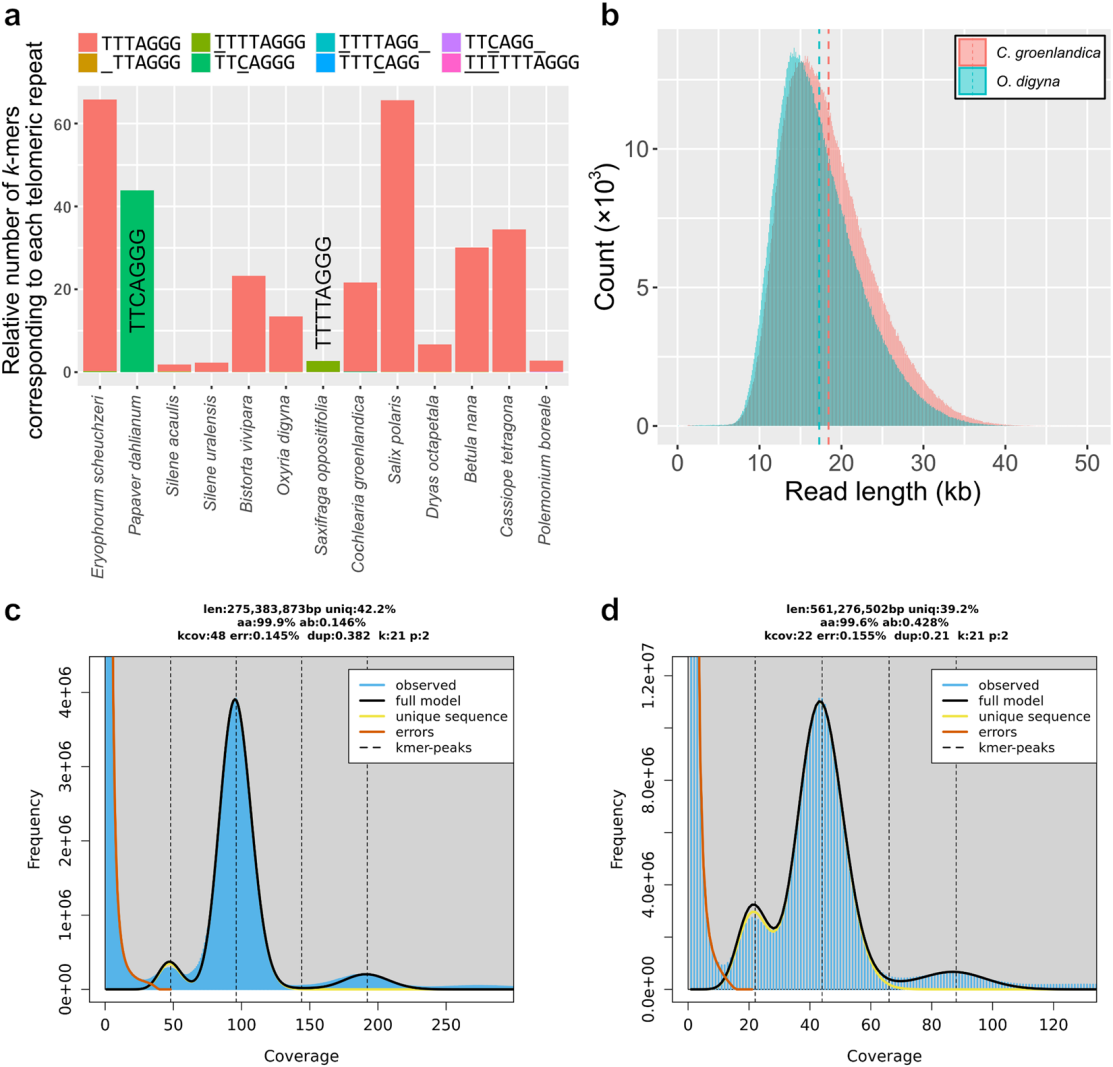
Genomic characteristics of the 13 plants and quality assessment of HiFi-based genome assemblies of *C*. *groenlandica* and *O*. *digyna*. (**a**) Relative number of telomeric-repeat *k*-mers that are previously known or newly identified in this study (TTCAGGG). Eight types of telomeric repeats were analyzed from the raw DNA sequencing reads of the 13 Svalbard plants. Different colors represent different telomeric repeats. In addition to the newly identified, TTCAGGG, the seven previously known telomeric repeats were counted: TTTAGGG (*Arabidopsis*-type; Richards and Ausubel 1988), TTAGGG (vertebrate-type; Weiss and Scherthan 2002; Sýkorová *et al*. 2003; Sýkorová *et al*. 2006), TTTTAGGG (*Chlamydomonas*-type; Petracek *et al*., 1990), TTCAGG (*Genlisea*-type; Tran *et al*., 2015), TTTCAGG (*Genlisea*-type; Tran *et al*., 2015), TTTTAGG (*Klebsormidium*-type; Fulnecková *et al*., 2013), and TTTTTTAGGG (*Cestrum*-type; Peška *et al*., 2015). (**b**) Raw HiFi read-length distribution of *C*. *groenlandica* and *O*. *digyna*. (**c,d**) Distributions of *k*-mer frequency found in the HiFi reads of *C*. *groenlandica* (**c**) and *O*. *digyna* (**d**). “len” represents the estimated genome size; “uniq” represents the ratio of unique sequences; “aa” represents the homozygosity rate; “ab” represents the heterozygosity rate; “kcov” represents the coverage for heterozygous *k*-mers; “err” represents the read error rate; “dup” represents the read duplication rate; “k” represents *k*-mer size used; “p” represents the ploidy level.

Possible telomeric repeats of every species were successfully discovered, and 11 out of 13 species contained canonical plant telomeric repeats, TTTAGGG (*Arabidopsis*-type) (Table 1 and Fig. 3a). The remaining two species, *P*. *dahlianum* and *S*. *oppositifolia*, had distinct noncanonical telomeric repeats. The telomeric repeat of *S*. *oppositifolia*, TTTTAGGG, is the *Chlamydomonas*-type^25^. However, the telomeric repeat of *P*. *dahlianum*, TTCAGGG, was novel.

### DNA and RNA sequencing analyses of *Oxyria digyna* and *Cochlearia groenlandica* for *de novo* genome assembly

All the experiments described in this section were performed by NICEM (https://nicem.snu.ac.kr). DNA was extracted using the cetyltrimethylammonium bromide (CTAB) method^34^. The library was prepared using the SMRTbell template prep kit 2.0 and sequenced on a PacBio Sequel IIe sequencer using the Sequel II sequencing kit 2.0. The computed data were subjected to the Circular Consensus Sequencing (CCS) protocol of the SMRT Link (ver. 11.0.0.146107) to derive the HiFi data (CCS reads of Q20 or higher). Based on the estimated genome sizes, we generated 25.5 Gb (1.5 M reads, 17 kb on average) and 30.2 Gb (1.6 M reads, 18 kb on average) of HiFi long-read sequencing data for *O*. *digyna* and *C*. *groenlandica* (Table 1 and Fig. 3b)^35,36^. HiFi reads were utilized to re-estimate their genome sizes using a *k*-mer-based method, KMC (version 3.2.1; *kmc -k21 -ci1 -cs1000000* and *kmc_tools transform histogram -cx1000000*) and GenomeScope2 (version 2.0; *genomescope.R --max_kmercov 1000000 -k 21*), with parameters optimized for highly repetitive genomes^37–41^. We estimated that *C*. *groenlandica* has ~275 Mb with a 0.15% heterozygosity and that *O*. *digyna* has ~561 Mb with a 0.43% heterozygosity (Fig. 3c,d). These estimated genome sizes differ from the initial ones estimated by KAT, but are much closer to the genome assembly sizes (see the next section).

Total RNA was extracted using the GeneAll Hybrid-RTM kit and RNA quality was measured using an Agilent Bioanalyzer RNA Nanochip. The library was prepared using the NEXTflex Rapid Directional RNA-Seq Bundle Kit (Perkin Elmer). qPCR was performed to quantify libraries capable of forming clusters and RNA sequencing was performed on the Illumina NovaSeq 6000 platform. We obtained 14 and 25.7 Gb (92.6 M and 170.3 M 151-bp paired-end reads) of RNA sequencing data for *O*. *digyna* and *C*. *groenlandica*, respectively (Table 1)^35,36^.

### *De novo* genome assembly and quality assessment using repetitive sequences and evolutionary conserved genes

For *de novo* genome assembly using HiFi sequencing data, data processing was conducted as described previously^42^. Specifically, we assembled 45 × and 121 × HiFi raw reads of *O*. *digyna* and *C*. *groenlandica*, respectively, into contigs using hifiasm (version 0.16.1; default option for *O*. *digyna* and *hifiasm -s 0.51* for *C*. *groenlandica*) and converted two GFA-formatted output files to FASTA-formatted files^43,44^. Previously known angiosperm repetitive sequences were masked using RepeatMasker (version 4.1.2.p1; *RepeatMasker -species angiosperms -s*) and identified *O*. *digyna*- and *C*. *groenlandica*-specific repetitive sequences using RepeatModeler (version 2.0.3; *BuildDatabase* and *RepeatModeler -database -LTRStruct -ninja_dir*) to mask species-specific repeats (version 4.1.2.p1; *RepeatMasker -lib -s*)^45^.

We further annotated repeats that were not classified by RepeatModeler using a transposon classification tool, RFSB (version 1.0; *transposon_classifier_RFSB -mode classify -fastaFile -outputPredictionFile*)^46^. The *Arabidopsis* type telomeric repeat, TTTAGGG, was searched among all ends of the contigs using RepeatMasker outputs, and the telomeric repeat clusters were manually validated^26^. To determine whether repeat density affected assembly quality, we calculated read depths and repeat compositions of 100-kb binned intervals of contigs ( ≥ 1 Mb for *C*. *groenlandica* and ≥ 10 Mb for *O*. *digyna* were used). We divided these intervals into the less repetitive or more repetitive groups using median repeat compositions of the intervals (median repetitive ratio: 0.57 for *C*. *groenlandica* and 0.69 for *O*. *digyna*). Their raw read depths were visualized as violin plots. BUSCO values were calculated using BUSCO and its Eudicot database (version 5.2.2; *busco -m genome --auto-lineage-euk*)^47,48^. Genome assembly quality values (QVs) and completeness were calculated based on *k*-mer using Merqury (version 1.3; *meryl k=21 count output* and *merqury.sh* with default option)^49^.

**Fig. 4:**
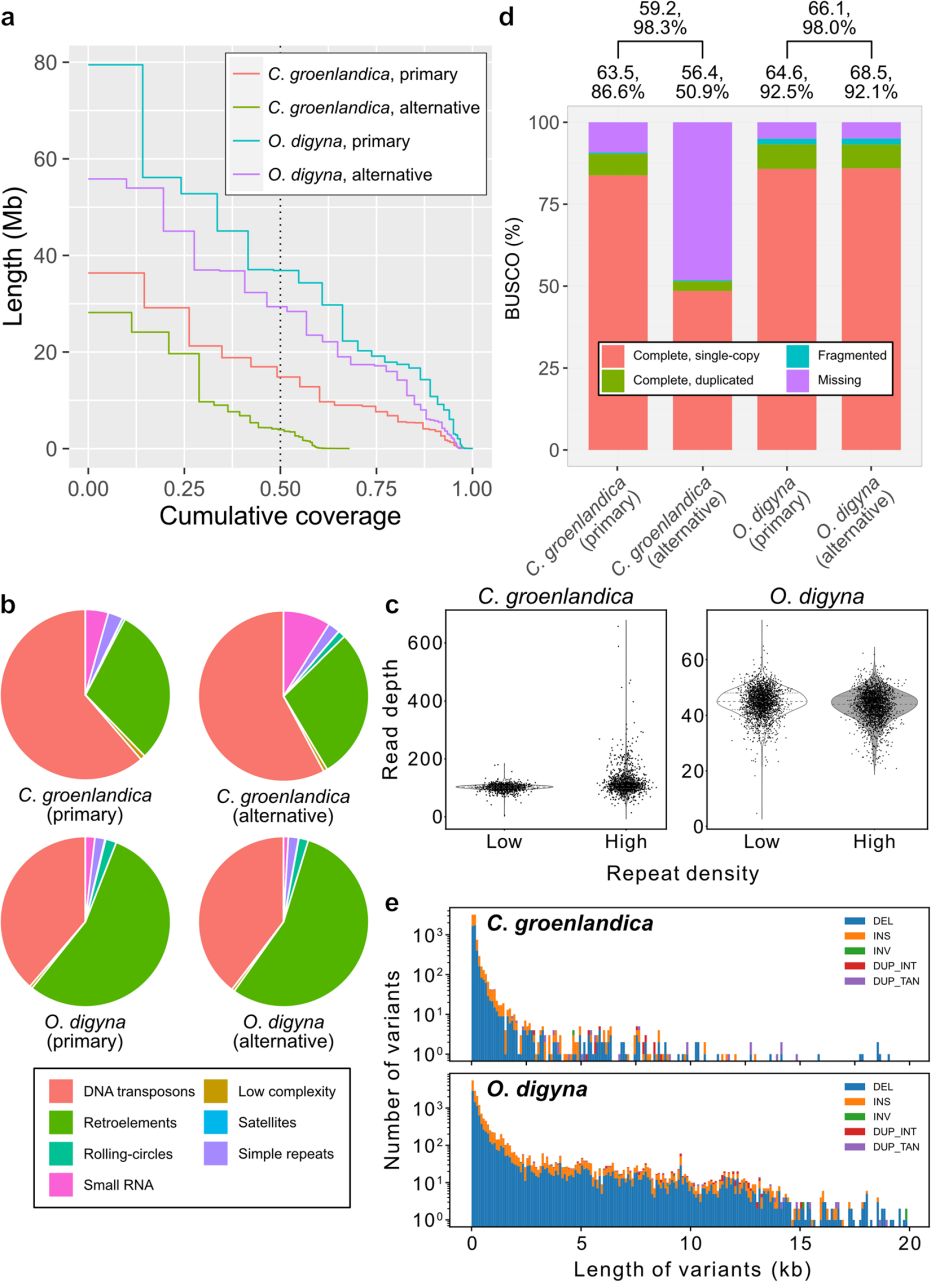
(**a**) Cumulative contig length distributions. The primary assembly sizes were used for the genome size for the two species to calculate NG50 (vertical dotted black line). (**b**) Repetitive sequence compositions in the genome assemblies. Numbers represent the lengths of each repetitive category. (**c**) Read depth distributions of less and more repetitive regions in *C*. *groenlandica* and *O*. *digyna* genome assemblies. Each dot represents the read depth of each 100-kb binned interval. (**d**) BUSCO values of each genome assembly (stacked bar graph). Numbers above the bars represent *k*-mer-based QV (upper) and completeness (lower) values. The top numbers represent QVs and completeness of merged genome assemblies for each species. (**e**) Length distributions of structural variants identified in *C*. *groenlandica* (upper panel) and *O*. *digyna* (lower panel) genome assemblies.

*De novo* genome assemblies using HiFi reads represented two partially phased haplotypes (primary and alternative) for both *O*. *digyna* and *C*. *groenlandica* (Fig. 4a and Table 2)^50,51^. The two genome assemblies had genome sizes of 561 Mb and 546 Mb for *O*. *digyna* and 250 Mb and 170 Mb for *C*. *groenlandica*. The longest contig lengths were 79.5 and 55.8 Mb for *O*. *digyna* and 36.4 and 28.2 Mb for *C*. *groenlandica*, and N50 lengths were 36.9 and 29.4 Mb for *O*. *digyna* and 14.8 and 9.0 Mb for *C*. *groenlandica*, respectively (Fig. 4a). These genome assembly sizes were concordant with the estimated genome sizes under the assumption of highly repetitive genomes (Fig. 3c,d).

**Table 2:** Summary statistics of draft genome assemblies.

Species	Genome assembly	Total size (Mb)	Maximum contig length (Mb)	Contig N50 length (Mb)	Number of contigs
*O*. *digyna*	Primary	561.29	79.47	36.88	359
*O*. *digyna*	Alternative	545.99	55.83	29.39	129
*C*. *groenlandica*	Primary	249.54	36.36	14.83	155
*C*. *groenlandica*	Alternative	169.63	28.18	8.99	481

Indeed, 69.0% (388 Mb) and 68.5% (374 Mb) of *O*. *digyna* and 61.9% (155 Mb) and 69.8% (118 Mb) of *C*. *groenlandica* were masked as repetitive sequences (Fig. 4b and Table 3). Approximately 96% (371 and 361 Mb) of *O*. *digyna* and 90% (142 and 104 Mb) of *C*. *groenlandica* repetitive sequences were classified as interspersed repeats, including DNA transposons and retroelements. As repetitive regions are difficult to be well assembled, we analyzed whether more repetitive regions exhibit more skewed read depth distributions by comparing read depths of less and more repetitive regions of the genome assemblies (Fig. 4c). In *C*. *groenlandica*, median read depths were similar in the two more and less repetitive regions (108.37 and 102.58, respectively), indicating that most repetitive regions were well resolved (Fig. 4c). However, some regions with high repeat density exhibited significantly high read depth (up to 6 times than the median), suggesting that some highly repetitive regions collapsed in our genome assembly (Fig. 4c). In *O*. *digyna*, the two regions showed much more similar read depth distributions and much lower variances than those of *C*. *groenlandica* (median: 45.04 for low and 44.00 for high; standard deviation: 5.02 for low and 5.34 for high). It indicates that repetitive regions in the *O*. *digyna* assembly were resolved as similar as non-repetitive regions. Moreover, the complete single-copy and complete duplicated BUSCO values of the assemblies were 93.3% and 93.2%, respectively, for the two *O*. *digyna* genome assemblies (eudicots_odb10) and 90.4% for the primary genome assembly of *C*. *groenlandica* (brassicales_odb10) (Fig. 4d), suggesting their high contiguity. However, the alternative genome assembly of *C*. *groenlandica* exhibited a complete BUSCO value of only 51.4%. This implies that it has many missing parts in its genome, concordant with its smaller genome size than that of the primary genome assembly (250 Mb vs. 170 Mb).

**Table 3:** Summary statistics for repetitive sequences (unit: Mb).

Type	*C*. *groenlandica*, primary	*C*. *groenlandica*, alternative	*O*. *digyna*, primary	*O*. *digyna*, alternative
Retroelement	46.43	34.09	212.52	206.07
DNA transposon	94.76	68.51	150.06	148.17
Rolling-circle	0.69	1.64	7.97	6.76
Small RNA	6.77	10.64	7.00	3.37
Satellite	0.07	0.04	0.57	0.20
Simple repeat	4.36	2.66	7.37	7.22
Low complexity	1.42	0.86	2.02	1.98

These genome assemblies were also assessed using *k*-mer-based QVs and completeness. *O*. *digyna* genome assemblies exhibited over QV60 and 92% completeness (Fig. 4d). In contrast, the primary genome assembly of *C*. *groenlandica* exhibited over QV60 and 87% completeness, but its alternative genome assembly exhibited only QV56 and 51% completeness (Fig. 4d). By merging primary and alternative genome assemblies for each species, their QVs were close to QV60, and their completeness was approximately 98% (Fig. 4d). It is noteworthy that the QVs were calculated using raw HiFi reads and HiFi-based genome assemblies, so the values could be overestimated, as they were not independently generated.

Overall, these metrics imply that our genome assemblies are highly accurate at the base level and mostly represent the full genome, except for the alternative genome assembly of *C*. *groenlandica*. We recommend only the primary genome assembly of *C*. *groenlandica*. These genome assemblies could be further scaffolded at the chromosome level in the near future with the advancement of other long-read and long-range sequencing technologies.

### Gene annotation and variant calling

We mapped RNA-seq raw reads to each soft-masked primary genome assembly of *O*. *digyna* and *C*. *groenlandica* using HISAT2 (version 2.2.1; *hisat2-build* and *hisat2*, default options) and sorted the output using SAMtools (version 1.11; *samtools sort* and *samtools index*)^52,53^. The output BAM file and soft-masked genome were used to annotate genes using BRAKER2 (version 2.1.6; *braker.pl --genome --bam --softmasking*)^52,54–57^. To identify known homologous genes, protein-coding genes were searched in the UniProt database (UniProtKB/Swiss-Prot Release 2021_03 of 02-Jun-2021; UniProtKB/TrEMBL Release 2021_03 of 02-Jun-2021) using MMseqs2 (version 9cc89aa594131293b8bc2e7a121e2ed412f0b931; *mmseqs easy-search -s 7*)^58,59^.

Single nucleotide polymorphisms (SNPs) were identified by mapping HiFi raw reads to the reference genome using minimap2 (version 2.22-r1101; *minimap2 -a -x map-hifi*); the mapping files were sorted using SAMtools (version 1.13; *samtools sort* and *samtools index*); and SNP calling was performed using DeepVariant (version 1.2.0; *run_deepvariant --model_type PACBIO --ref --reads --output_vcf*)^52,60–62^. SNPs of genes were analyzed using SnpEff (version 5.1d; *java -jar snpEff.jar build -gtf22 -v DBname* and *java -jar snpEff.jar DBname VCFfile*)^63^. To identify structural variants (SVs), we first aligned our alternative assembly to the reference using Winnowmap2 (version 2.03; *meryl count k*=*19*, *meryl print greater-than distinct*=*0.9998*, and *winnowmap -W -ax asm20 --cs -r2k*) and sorted the output using SAMtools (version 1.7; *samtools sort* and *samtools index*)^52,64^. The output BAM file was analyzed using SVIM-asm to call the SVs (version 1.0.2; *svim-asm haploid*)^65^. Genes affected by SVs were analyzed using BEDTools (version v2.30.0; *bedtools intersect*)^66,67^.

We identified 43,105 and 29,675 possible protein-coding genes in *O*. *digyna* and *C*. *groenlandica*, respectively, of which 33,134 and 27,381 were searched in the UniProt database (Table 4). By mapping raw HiFi reads onto our reference genome, we identified 765,012 and 88,959 heterozygous SNPs comprising 0.14% and 0.04% of the total genomic length, respectively (Table 4). The effect of SNPs on genes were categorized as follows: 1,001 high-impact, 22,592 moderate-impact, and 15,594 low-impact SNPs for *O*. *digyna* and 253 high-impact, 6,536 moderate-impact, and 6,373 low-impact SNPs for *C*. *groenlandica* (Table 4). We found 9,574 deletion SVs, 8,302 insertion SVs, 26 inversion SVs, 97 interspersed duplication SVs, and 83 tandem duplication SVs in *O*. *digyna* and 4,079 deletion SVs, 3,577 insertion SVs, 5 inversion SVs, 10 interspersed duplication SVs, and 27 tandem duplication SVs in *C*. *groenlandica* (Table 4 and Fig. 4e). Coding sequences in 1,104 and 328 genes of *O*. *digyna* and *C*. *groenlandica*, respectively, were affected by SVs. Of these, 747 and 147 genes had known homologs (Table 4).

**Table 4:** Summary of numbers of genes and genetic variants.

Species	*C*. *groenlandica*	*O*. *digyna*
Protein-coding genes	29,675	43,105
Known homologs	27,381	33,134
SNP	88,959	765,012
SNP ratio compared to the genome size (%)	0.04	0.14
High-impact SNP	253	1,001
Moderate-impact SNP	6,536	22,592
Low-impact SNP	6,373	15,594
Deletion SV	4,079	9,574
Insertion SV	3,577	8,302
Inversion SV	5	26
Interspersed duplication SV	10	97
Tandem duplication SV	27	83
SV ratio compared to the genome size	0.003	0.003
Genes affected by SV	328	1,104
Genes with known homologs & affected by SV	147	747

### Circos visualization

The numbers of genes, genetic variants, and repetitive sequences were summed for every 100 kb bin by utilizing the output files of BRAKER2, DeepVariant, SVIM-asm, and RepeatMasker. HiFi read depths for every position were obtained by applying SAMtools to the intermediate BAM files of the DeepVariant analysis (version 1.13; *samtools depth -a*) and were summed for every 100 kb bin^52^. We visualized the gene, genetic variant, repeat, and read depth densities using Circos (version v 0.69-8; *circos -conf configurationFile.txt*; *plot type = histogram* for read depths and *plot type heatmap* for gene, genetic variant, and repeat densities)^68^ (Fig. 2).

## Data Records

All sequencing reads generated in this study have been deposited in the NCBI Sequence Read Archive under accession numbers, SRP404573, SRP427161, and SRP468445^21,35,36^. The assemblies have been deposited in GenBank under the accession numbers, GCA_029168935.1 and GCA_040259375.1^50,51^. Genome sequences, gene annotation, and amino acid and coding sequences of annotated genes are available at figshare database^69^. Homology search and variant impact analysis for genes and repeat classification data are also available at figshare^69^.

## Technical Validation

Illumina DNA sequencing reads and PacBio HiFi DNA sequencing reads were produced > 25 Gb. HiFi reads showed qualified read length distributions (mean read length: 17,289 and 18,429 bp for *Oxyria digyna* and *Cochlearia groenlandica*, respectively). Assembly quality was assessed using contig length and BUSCO. The N50 lengths of the primary assemblies were greater than 10 Mb, and the longest contig lengths were 79 and 37 Mb, respectively. BUSCO values exceeded 90%.

## Data Availability

All scripts, parameters, and options used in the study are described in the Methods. We used publicly available programs and not custom programs.
